# Cerebral Oxygenation During Neonatal Intubation–Ancillary Study of the Prettineo–Study

**DOI:** 10.3389/fped.2019.00040

**Published:** 2019-03-01

**Authors:** Meryl Vedrenne-Cloquet, Sophie Breinig, Agnes Dechartres, Camille Jung, Sylvain Renolleau, Laetitia Marchand-Martin, Xavier Durrmeyer

**Affiliations:** ^1^Neonatal Intensive Care Unit, CHI Créteil, Créteil, France; ^2^Pediatric Intensive Care Unit, Necker University Hospital, Paris, France; ^3^Neonatal and Pediatric Intensive Care Unit, Toulouse University Hospital, Toulouse, France; ^4^Inserm U1136, Institut Pierre Louis d'Epidémiologie et de Santé Publique, Département Biostatistique santé publique, information médicale–Hôpital Pitié Salpêtrière, Assistance Publique-Hôpitaux de Paris (APHP), Sorbonne Université, Paris, France; ^5^Clinical Research Center, CHI Créteil, Créteil, France; ^6^INSERM, UMR1153, Obstetrical, Perinatal and Paediatric Epidemiology (Epopé) Team, Epidemiology and Biostatistics Sorbonne, Paris Descartes University, Paris, France; ^7^Faculté de Médecine de Créteil, IMRB, GRC CARMAS, Université Paris Est Créteil, Créteil, France

**Keywords:** neonatal intubation, premedication, cerebral oxygenation, near infrared spectroscopy, hypotension, propofol

## Abstract

**Purpose:** This study aimed to describe cerebral Near InfraRed Spectroscopy (NIRS) profiles during neonatal intubation using two different premedication regimens.

**Methods:** Neonates requiring non-emergency intubation were enrolled in an ancillary study, conducted in two French Neonatal Intensive Care Units participating in a larger on-going multicenter, double blind, randomized, controlled trial. Patients were randomly assigned to the “atropine-propofol” (Prop) group or the “atropine-atracurium-sufentanil” (SufTrac) group. Regional cerebral oxygen saturation (rScO_2_), pulse oxymetry (SpO_2_), mean arterial blood pressure (MABP), and transcutaneous partial pressure of carbon dioxide (TcPCO_2_) were collected at 9 predefined time points from 1 min before to 60 min after the first drug injection. The two primary outcomes were a decrease in rScO_2_ value >20% from baseline and a decrease in fractional cerebral tissue oxygen extraction (FTOE) value >10% from baseline, at any time point. Secondary outcomes included physiological parameters changes over time and correlations between mean arterial blood pressure, and FTOE at different time points. Descriptive results were obtained and exploratory statistical analyses were performed for 24 included patients.

**Results:** rScO_2_ decreased in 5/11 (46%) infants from the Prop group and 10/13 (77%) from the SufTrac group (*p* = 0.11); FTOE decreased in 10/11 (91%) infants from the Prop group, and 12/13 (92%) from the SufTrac group (*p* = 0.90). rScO_2_ values decreased over time in both groups, whereas FTOE's pattern appeared more stable. SpO_2_ and transcutaneous TcPCO_2_ seemed more preserved in the Prop group while MABP seemed more preserved in the SufTrac group. No important correlation was observed between MABP and FTOE (r = 0.08 to 0.12 across the time points).

**Conclusion:** Our results suggest a frequent decrease in cerebral oxygenation without obvious impairment in cerebral autoregulation during neonatal intubation with premedication. This study confirms the feasibility and the informative value of cerebral NIRS monitoring in this setting.

**Clinical Trial Registration:** www.ClinicalTrials.gov, identifier NCT02700893.

## Introduction

Premedication is recommended before a (semi)-elective neonatal intubation (1) because awake intubation is painful and increases intracranial pressure (2). However, awake intubation remains common in some Neonatal Intensive Care Units (NICU) (3, 4), partly because of the fear of adverse drug reactions (4). The combination of a short-acting opioid with a muscle-blocker is among recommended regimens for premedication prior to neonatal intubation (1, 5) but can lead to thoracic rigidity and hypoxemia in very preterm infants (6). Propofol is a short-acting anesthetic that preserves spontaneous ventilation (7, 8) but can cause systemic hypotension (8, 9).

Low systemic blood flow is associated with brain damage in premature neonates (10, 11) since their cerebral autoregulation (CAR) is frequently impaired (12, 13). However, systemic hypotension, defined as a mean arterial blood pressure (MABP) (in mmHg) lower than gestational age (in weeks) (14), is poorly associated with low systemic blood flow (15). A multimodal evaluation has been proposed to assess cerebral hemodynamics and oxygenation beyond blood pressure monitoring (16), using Near Infra Red Spectroscopy (NIRS) (17). This technology offers a non-invasive monitoring of peripheral microcirculation, reflecting the adequacy between oxygen delivery and consumption *in situ* (18). Cerebral NIRS is easily achieved in neonates, allowing measurements of cerebral oxygen saturation (rScO_2_) without serious adverse effects (19), and has been found a useful tool during laryngoscopy (20–23).

This study aimed to describe cerebral oxygenation assessed by NIRS during neonatal intubation with 2 different premedication regimens: atropine-propofol or atropine-atracurium-sufentanil.

## Methods

### Study Design and Setting

We conducted a prospective observational ancillary study strictly following the design and methods of a previously published multicenter, double-blind, randomized, controlled trial with parallel groups (24). The only particularity of this ancillary study was to collect cerebral NIRS values during the patients' participation in the study. This ancillary study was conducted in two of the 6 participating centers that had previous experience and available equipment for NIRS monitoring. This ancillary study was conceived in September 2015, after the main trial's interruption in August 2014. This ancillary study started in March 2016 when the main trial was resumed (see Supplement) (24). The ancillary study and the main trial were simultaneously prematurely interrupted in August 2016 for expired study kits and unavailability of additional funding (24).

Briefly, in this trial, neonates hospitalized in the NICU requiring a non-emergency endotracheal intubation were randomly assigned 1:1 to the “atropine-propofol” (Prop) group or the “atropine-atracurium-sufentanil” (SufTrac) group using a fixed block size of 4, and stratification by center and weight (≤ or > 1,000 g). The premedication and intubation procedures have already been reported elsewhere (24) and are detailed and illustrated in the Supplemental Figure 1. A double-dummy approach was used with intralipids as a placebo for propofol and normal saline as a placebo for atracurium and sufentanil so that treatments appeared identical (Supplemental Figure 1). Parents, physicians and, nurses were unaware of treatment allocation. The primary outcome of the main trial was prolonged desaturation.

### Participants

All patients included in the main trial in the two centers between March and August 2016, were eligible for the ancillary study. Inclusion criteria were exactly similar to those of the main study and included: corrected age at the time of inclusion of < 45 weeks (in order to recruit exclusively neonates defined as infants with a maximal postnatal age of 28 days after full-term birth, which corresponds to 45 weeks of corrected age) and indication for non-emergency intubation. Exclusion criteria were exactly similar to those of the main study and included: sedatives or anesthetics administration in the previous 24 h, hemodynamic failure (MABP less than corrected gestational age and/or capillary refill time exceeding 3 s), upper airway malformation, life-threatening situation, previous inclusion in this, or in another trial, any contraindication to any study drug. In this ancillary study the only additional exclusion criteria was the presence of skin lesions on the forehead that precluded the use of NIRS sensors.

### Measurements and Outcomes

Data related to NIRS cerebral profiles were collected during the procedure using a 2-wavelength (730–810 nm) cerebral oxymeter (monitor INVOS 5100C®, Covidien, Medtronics, USA) to record rScO_2_. A transducer (neonatal sensor OXYALERT, Covidien, Medtronics, USA) was placed on the left fronto-parietal side of the patient's head. Pulse oxymetry (SpO_2_) acquired with an oximetry sensor placed on the right hand (LNOP® Neo-Pt L or Newborn, Masimo INC., Irvine, CA), transcutaneous partial pressure of carbon dioxide (TcPCO_2_), non-invasive blood pressure, and heart rate (HR) were also collected (24). Data were collected 1 min before the first drug injection, at the first drug injection, and at 3, 6, 9, 12, 15, 30, 45, and 60 min after it. To assess the balance between oxygen delivery and consumption, the Fractional cerebral Tissue Oxygen Extraction (FTOE) was calculated from rScO_2_ and pulse oxymetry (SpO_2_) as this ratio: FTOE = [SpO_2_-rScO_2_]/SpO_2_ (25, 26) for all time points.

The two predefined primary outcomes were a cerebral desaturation, defined as a decrease in rScO_2_ value > 20% from baseline (27, 28), and an impaired cerebral oxygen extraction, defined as a decrease in FTOE value > 10% from baseline, at any time point after the first injection. No consensual threshold exists to define a significant decrease in FTOE, so we arbitrarily set this threshold at 10% since a previous study considered a 5% decrease “small and probably clinically irrelevant” (29).

Secondary outcomes included changes over time for HR, MABP, SpO_2_, TcPCO_2_, rScO_2_, and FTOE in both groups. As a surrogate for CAR, we studied the correlation between MABP and FTOE at different predefined time points (1 min before and 6 min, and 15 min after the first injection), considering that the frequent occurrence of systemic desaturation during intubation (24) would preclude the interpretation of correlations between MABP and rSCO_2_ (17, 23).

### Statistical Analysis

This ancillary study was designed to provide observational data. No hypothesis was formulated. Thus, we did not conduct a formal sample size calculation for this ancillary exploratory study.

Results are summarized as medians and IQR for quantitative variables, and numbers and percentages for qualitative variables. We took into account the limited sample size and the obvious imbalance in some characteristics between groups. Therefore, baseline characteristics were compared between treatment groups, using Mann-Whitney and Fisher's exact tests or continuous and categorical variables, respectively, in spite for current recommendations concerning randomized trials (30) Due to the small sample size, we performed exploratory comparative tests using Fisher's exact tests for the primary outcomes. A *p* < 0.05 was considered statistically significant.

To analyze the link between MABP and FTOE, a Pearson correlation coefficient was calculated at predefined time-points (1 min before and 6 min and 15 min after the first injection).

No imputation of missing data was performed. Data were analyzed using SAS ® 9.4.

## Results

### Study Population

**Figure 1:**
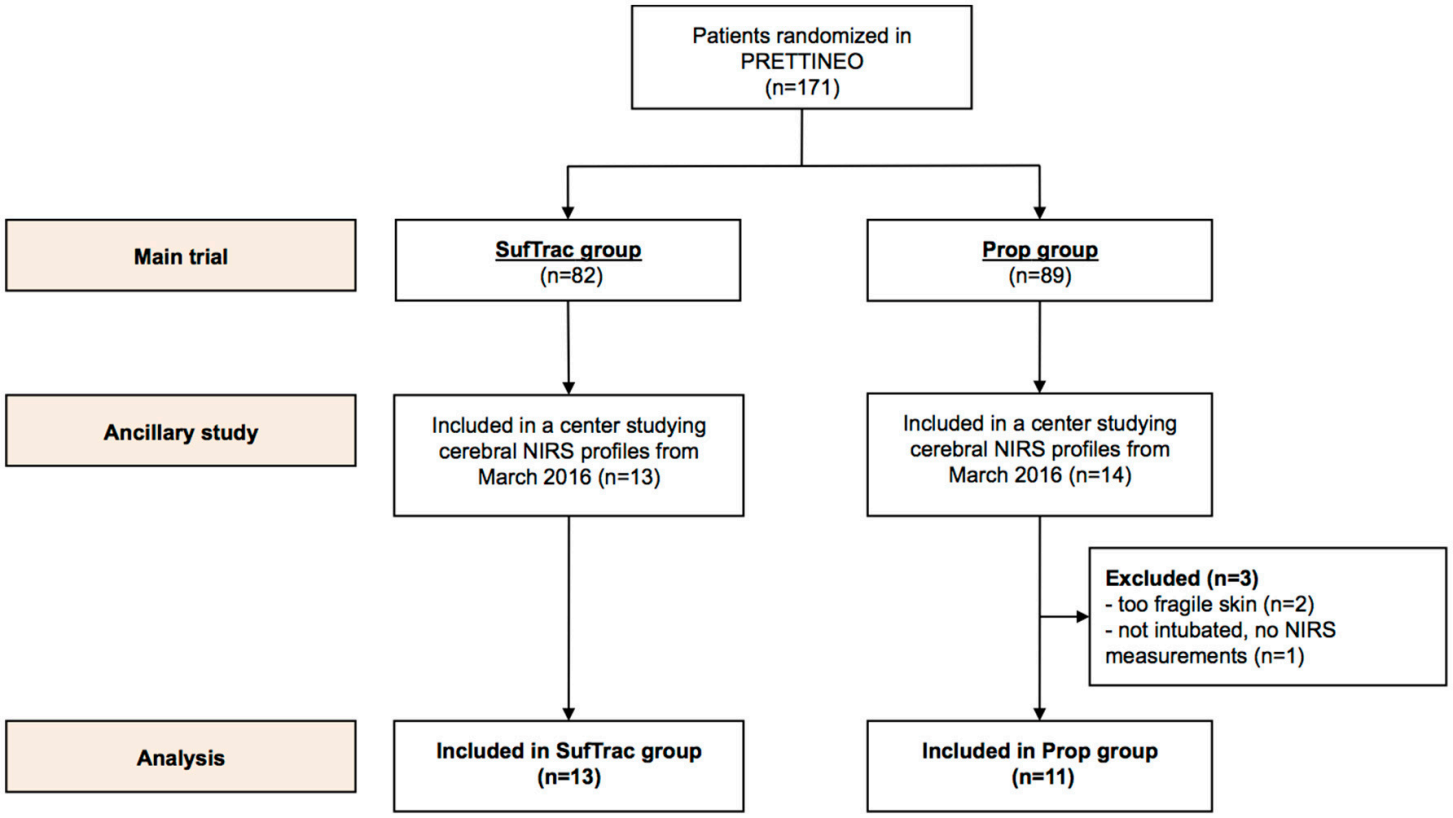
Population flow chart.

Between 1 March 2016 and 31 August 2016, 27 infants were included in the main study in the two centers participating in the ancillary study (flow chart presented in Figure 1). Three patients allocated to the Prop group were excluded because NIRS measurements could not be performed. Data were collected and analyzed in 24 patients: 11 in the Prop group and 13 in the SufTrac group.

Clinical characteristics of patients from each group are provided in Table 1. Despite randomization, gestational age, and weight at inclusion were lower in the SufTrac group than in the Prop group. Baseline physiological and NIRS parameters were comparable (Table 1). In the SufTrac group, 12/13 patients received non-invasive ventilation before intubation. In the Prop group, 6/11 patients received non-invasive ventilation before intubation, and 4/11 patients were spontaneously breathing room air before intubation. These four patients were intubated in order to perform planned surgery under general anesthesia. One patient in each group was receiving invasive ventilation before the procedure and underwent endotracheal tube change.

**Table 1:** Population baseline characteristics.

	**SufTrac group (*n* = 13)**	**Prop group (*n* = 11)**	
	**Median [IQR] or *n* (%)**	**Median [IQR] or *n* (%)**	***p*-value^†^**
Gestational Age at birth, weeks	28.0 [26.0; 32.0]	34.0 [30.0; 38.0]	0.042
Birth weight, g	1120.0 [800.0; 1480.0]	1815.0 [1505.0; 3250.0]	0.018
Male	9 (69.2)	6 (54.5)	0.68
Five-min Apgar score	9.0 [8.0; 10.0]	9.0 [8.0; 10.0]	0.72
Age at inclusion, days	0.0 [0.0; 5.0]	1.0 [0.0; 8.0]	0.78
Parameters at T-1 min			
HR, bpm	151.0 [142.0; 155.0]	157.0 [138.0; 167.0]	0.30
Systolic arterial	59.0 [53.0; 65.0]	57.0 [49.0; 68.0]	0.93
pressure, mmHg			
Diastolic arterial	34.0 [30.0; 49.0]	31.0 [24.0; 41.0]	0.54
pressure, mmHg			
MABP, mmHg	40.0 [38.0; 54.0]	40.0 [36.0; 51.0]	0.64
SpO_2_, %	98.0 [96.0; 99.0]	98.0 [98.0; 100.0]	0.31
TcPCO${}_{2}^{*}$, mmHg	48.5 [42.5; 65.5]	53.0 [39.0; 61.5]	0.67
rScO_2_, %	72 [67; 85]	77 [71; 89]	0.28
Ventilatory support at T-1 min			0.044
Mechanical	1 (7.7)	1 (9.1)	
ventilation			
Non-invasive	12 (92.3)	6 (54.6)	
ventilation			
Spontaneous	0 (0)	4 (36.4)	
FiO_2_, %	50 [35; 100]	51 [21; 99]	0.43
ventilation			
Previous intubation	5 (38.5)	2 (18.2)	0.39
Reason for intubation			0.021
Respiratory distress	10 (76.9)	5 (45.5)	
Apnea	2 (15.4)	0 (0)	
Surgery	0 (0)	5 (45.5)	
Endotracheal tube	1 (7.7)	1 (9.1)	
change			

*T-1 min defines the baseline value (first value recorded 1 min before drug injection)*.

*n = 8/group.

† *Mann-Whitney test for continuous variable or exact Fisher test for categorical variable*.

*bpm, beat per minute; n, number of subjects; IQR, Interquartile Range; SufTrac, Atropine-Atracurium-Sufentanil; Prop, Atropine-Propofol; HR, Heart Rate; MABP, Mean Arterial Blood Pressure*.

### Interventions Received

Patients in the Prop group received more often additional treatment as planned in the protocol (7/11, 64%) than patients in the SufTrac group (2/13, 15%). In the Prop group, among the 7 patients who received 6 syringes, 4 received an additional open-label treatment: 2 received atracurium and sufentanil, one received propofol and atracurium, and one received propofol alone. The median [IQR] cumulated propofol dose in this group was 3.0 [2.5–3.5] mg/kg. In the SufTrac group, no infant required open label treatment.

### Primary Outcomes

A cerebral desaturation occurred in 5/11 patients (46%) from the Prop group and in 10/13 patients (77%) from the SufTrac group (*p* = 0.11). A decrease in FTOE occurred in 10/11 patients (91%) from the Prop group and in 12/13 patients (92%) from the SufTrac group (*p* = 0.90).

### Physiological Parameters and Cerebral Oxygenation Changes Over Time

**Figure 2:**
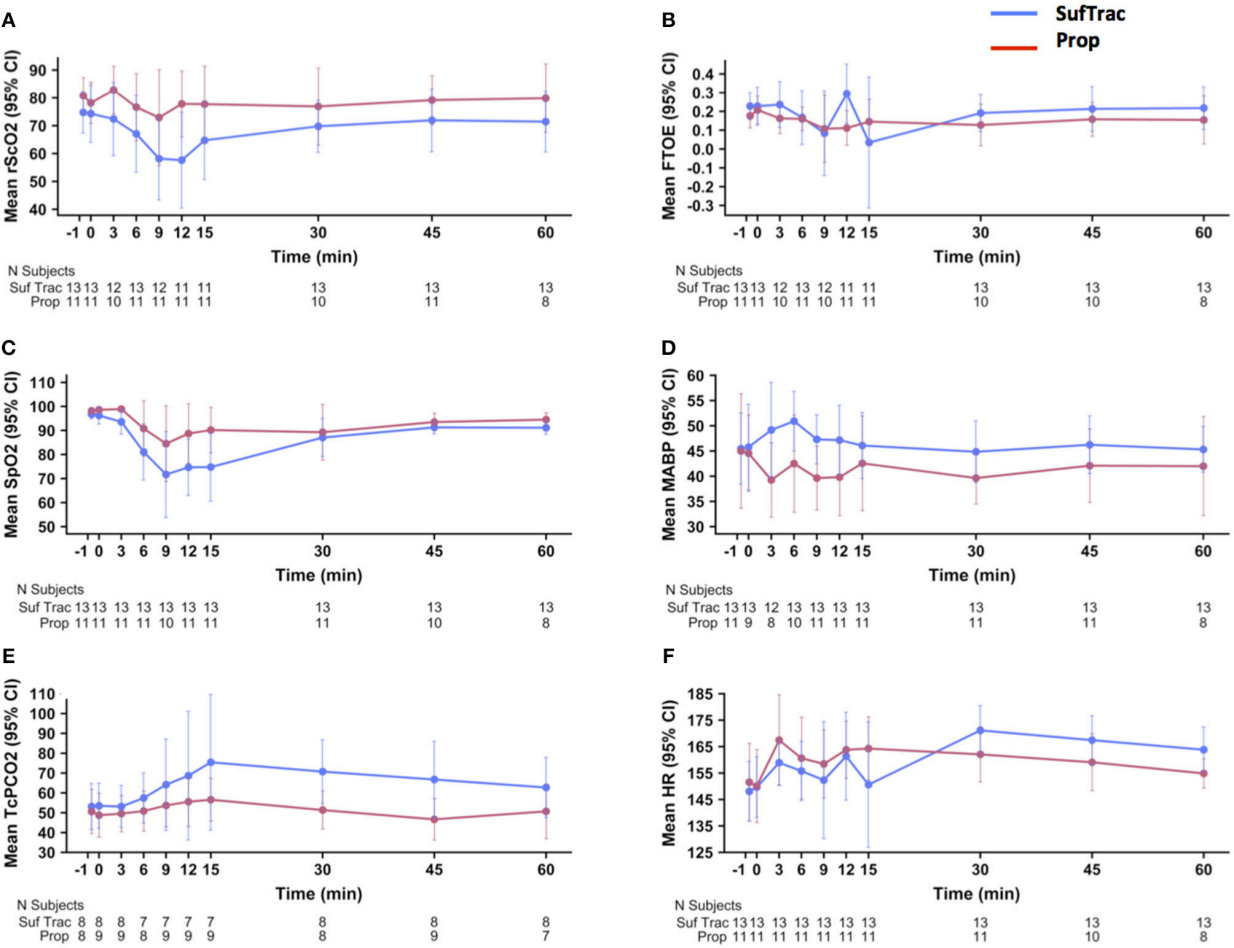
Physiological parameters and cerebral oxygenation changes overtime. These graphs illustrate the mean values (points) and 95% CI (error bars) on the Y axis for rScO_2_ **(A)**, FTOE **(B)**, SpO_2_ **(C)**, MABP **(D)**, TcPCO_2_ **(E)**, and HR **(F)** over time. The x-axis indicates the time before and after first drug injection (denoted as “0”) in min. Graphs are presented in red for the atropine-propofol group and in blue for the atropine-atracurium-sufentanil group. CI, confidence interval; rScO_2_, regional cerebral oxygen saturation; FTOE, fractional cerebral tissue oxygen extraction; SpO_2_, oxygen saturation; MABP, mean arterial blood pressure; TcPCO_2_, transcutaneous partial carbon dioxide pressure; HR, heart rate.

Figure 2 shows the mean rScO_2_, FTOE, SpO_2_, MABP, TCpCO_2_, and HR values over time in each group. rScO_2_ decreased in both groups at 6, 9, and 12 min after the first injection, with subsequent return to baseline and a graphically deeper decrease in the SufTrac group (Figure 2A). Mean SpO_2_ decreased at 6, 9, 12, and 15 min after the first injection, with subsequent return to values > 90% after 30 min and a graphically deeper decrease in the SufTrac group (Figure 2C). Mean MABP decreased in the Prop group, and initially increased then returned to baseline in the SufTrac group (Figure 2D). TcPCO_2_ remained stable in the Prop group and increased in the SufTrac group (Figure 2E). Heart rate increased in both groups (Figure 2F). Although a 10% decrease in FTOE at any time point was frequent in both groups, the mean FTOE pattern over time remained relatively stable during monitoring with remarkably superimposable curves in both groups (Figure 2B).

### FTOE/MABP Correlation

**Figure 3:**
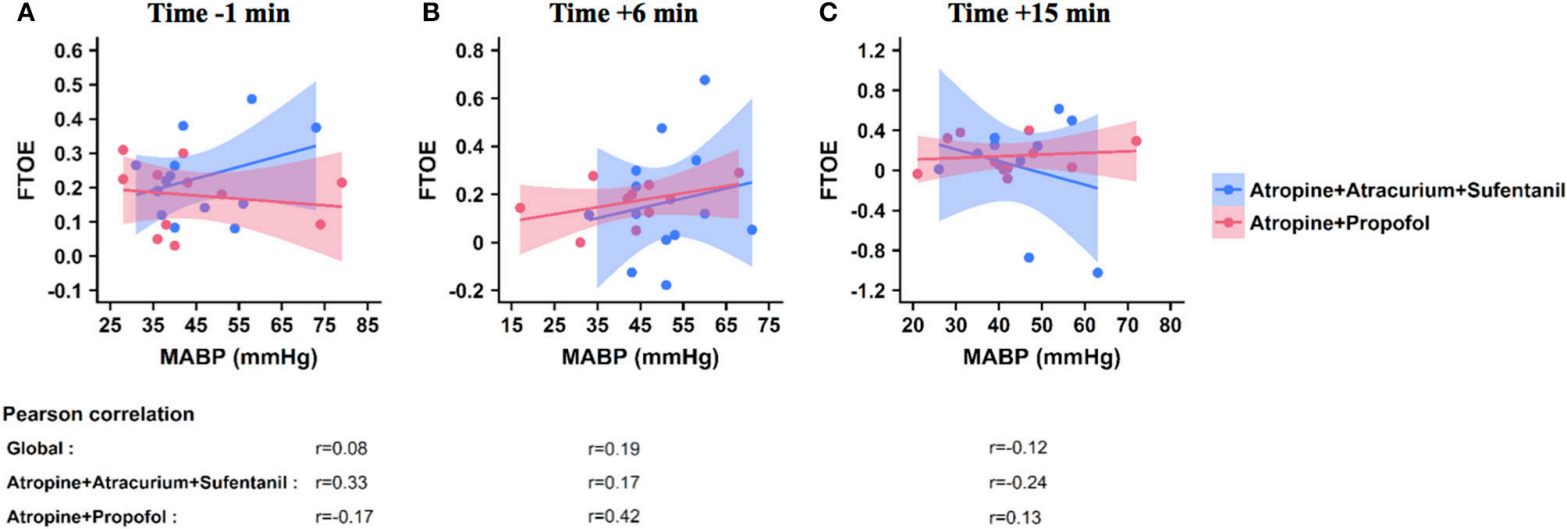
Correlation between FTOE and MABP at predefined time points. These graphs illustrate the correlation between FTOE (Y axis) and MABP (X axis) 1 min before **(A)**, and 6 min **(B)**, and 15 min **(C)** after the first drug injection. Each point represents a patient, the solid line represents the regression line and the colored area represents the 95% CI interval within each group (atropine-propofol in red, atropine-atracurium-sufentanil in blue). Pearson correlation coefficients calculated for the whole population (global) and for each group are included below the graph for each time point. FTOE, fractional cerebral tissue oxygen extraction; MABP, mean arterial blood pressure.

Correlations between FTOE and MABP are shown in Figure 3. No correlation higher than 0.5 was found between FTOE and MABP 1 min before (Figure 3A), 6 min (Figure 3B), or 15 min (Figure 3C) after the first injection in both groups. Correlation coefficients varied between 0.08 and 0.19 in the whole population, between 0.17 and 0.33 in the SufTrac group, and between 0.13 and 0.42 in the Prop group (Figure 3).

## Discussion

To our knowledge, this is the first study reporting cerebral NIRS profiles with two premedication regimens during neonatal intubation. This study demonstrated the feasibility of cerebral oxygenation monitoring during this procedure. rSCO_2_ decreased over time and FTOE frequently decreased from baseline but remained relatively stable over time. FTOE curves were graphically superimposable in the two treatment groups. FTOE was not strongly correlated with MABP.

Strengths of this study include the quality of evidence due to the design of the main study and its originality since cerebral oxygenation profiles were described with 2 premedication regimens acceptable for neonatal intubation. The findings provide limited but valuable information on the studied drugs' safety and should discourage physicians from performing awake neonatal intubation outside life-threatening situations.

To date, three studies have analyzed cerebral NIRS profiles during neonatal intubation. van den Berg et al. compared infants receiving an InSurE (Intubation-Surfactant-Extubation) procedure preceded by morphine or pethidin to matched controls with nCPAP (20). Bertini et al. compared cerebral NIRS profiles during surfactant administration either by an InSurE or a LISA (Less Invasive Surfactant Administration) technique, both requiring a laryngoscopy, without premedication (21). Smits et al. conducted a dose-finding study on propofol used as premedication before a (semi)-elective intubation (22) and specifically assessed CAR in a subset of this population in another publication by Thewissen et al. (23). Although Van den Berg et al. found no statistically significant effect of the InSurE procedure on rScO_2_ or FTOE (20), the other studies found a short-lasting decrease in rScO_2_ just after the procedure (21–23), which is consistent with our results. Whereas, mean FTOE values seemed more stable over time in our study, Bertini et al. found an increase in FTOE after the LISA procedure but not after the InSurE procedure. This finding was interpreted as a “compensatory mechanism” but no speculation was made on CAR (21). Conversely, Smits et al. found a decrease in FTOE after intubation with propofol, that they attributed to a decrease in cerebral consumption (22).

In our study, cerebral desaturation was concomitant with systemic desaturation, especially in the SufTrac group. Systemic desaturation leads physiologically to cerebral desaturation. Oxygen transport to the living tissues depends on the arterial oxygen content, which is determined among others by arterial oxygen saturation and hemoglobin. Thus, a decrease in rScO_2_ can occur in case of an isolated hypoxemia without hemodynamic disturbance. In stable preterm babies, multiple episodes of hypoxemia alone can lead to a decrease in rScO_2_ without any trouble in cerebral FTOE (31). Smits et al. also described this phenomenon and defined it as a “hypoxic hypoxia” rather than an “ischemic hypoxia,” because of a brief and short-lasting decrease in rScO_2_ despite a prolonged decrease in MABP (22). Our results are consistent with this hypothesis since the decrease in SpO_2_ was concomitant with the decrease in rScO_2_. Moreover, FTOE values were preserved in the two groups despite a low rScO_2_. As FTOE reflects the balance between oxygen demand and supply, this suggests a trouble affecting the cerebral oxygen delivery without any trouble in oxygen consumption by the brain tissue. This might be attributable to a physiological process called “neurometabolism coupling,” which links cerebral blood flow and vasoreactivity to cerebral metabolic demand. Cerebral oxygen consumption reflects cerebral metabolism, which depends on cerebral oxygen extraction and cerebral blood flow. We can assume that in our study, patients had low cerebral oxygen consumption induced by sedative drugs; this could have led to a lower cerebral blood flow that maintained a stable FTOE despite a low rScO_2_. This hypothesis is supported by the results from the ancillary study of the NEOPROP study that found mostly intact CAR with suppressed cerebral activity measured by aEEG in preterm infants premedicated with propofol for an INSURE procedure (23).

Some physiological parameters, such as SpO_2_ or MABP seemed to have different patterns over time in the 2 treatment groups. These seemingly different observed patterns could be attributed to many factors such as the treatment group, the imbalance between groups in patients' weight, gestational age, and indications for intubation, or chance. Thus, the interpretation of these results should be careful.

Deeper systemic desaturation observed in the SufTrac group might also be due to the decrease in lung residual capacity due to the use of a muscle-blocker (atracurium) (6), while propofol is known to preserve spontaneous ventilation. The higher TcPCO_2_ in this group is consistent with this hypothesis and was previously reported in other studies using muscular-blockers prior to neonatal intubation (6, 32).

MABP seemed to be more preserved in the SufTrac group than in the Prop group. Despite decreased MABP in the Prop group, FTOE values from both groups were superimposable and no strong correlation was found between FTOE and MABP. Results in the Prop group support the existing hypothesis that a drop in tissue oxygenation is uncertain during propofol-induced hypotension (22, 23, 33, 34). A correlation coefficient < 0.5 has been considered to rule out impaired CAR (35). In our study, we observed low correlation coefficient, suggesting that most of our population had a preserved CAR. Consistently, Thewissen et al. observed one case among 22 patients of impaired CAR associated with systemic hypotension, although 5 other cases of impaired CAR were observed with normal or elevated MABP (23). Based on these results isolated hypotension with signs of good perfusion might be respected (16). However, our findings might not be applicable to critically ill neonates such as extremely low birth weight neonates (13) or infants who had non-inclusion criteria for our study, such as needing emergency intubation or having signs of hemodynamic compromise.

Our study had two major limitations. First, patients were recruited only between March and August 2016, while the larger trial started in 2011, resulting in a limited number of recruited patients. Second, there were important imbalances in gestational age and weight at inclusion, and in indication for intubation between groups, leading to different populations despite randomization. This might have biased results, although baseline characteristics concerning physiological parameters including rScO_2_ were comparable at inclusion. However, the patterns of physiological parameters' changes over time in the recruited patients were very similar to those observed in the main trial (24), suggesting a representative sample. The third limitation of our study is its exploratory nature, without any pre-specified hypothesis concerning a specific NIRS-related outcome, nor sample size calculation. Our study may therefore lack power and potentially expose to selection bias. These first three limitations did not allow us to perform a formal comparison between the 2 studied regimens so we cannot conclude about any differences between them. Fourth, for MABP measures we used non-invasive measures that might not be correlated with invasive measures in premature neonates (36). Fifth, we analyzed correlations between FTOE and MABP at predefined time points and did not collect data continuously as others (23). Thus, we could not assess coherence, which is a better estimation of CAR (23, 37).

Cerebral NIRS appeared as a feasible and informative monitoring device to investigate the hemodynamic tolerance of neonatal intubation with premedication. For both premedication regimens a decrease in rSCO_2_ that paralleled a decrease in SpO_2_ was observed during neonatal intubation, but the study's design did not allow for comparison between the premedication regimens. However, no strong indicator of impaired CAR was observed. Adequately powered trials using cerebral NIRS are required to further assess the safety of premedication drugs for neonatal intubation.

## Ethics Statement

The study was approved by an ethics committee (Paris Ile de France 3, approval n° 2895) and by the French Medicinal Products Agency (n° A110281-16). Written informed consent was obtained from the parents of all infants.

## Author Contributions

MV-C and XD conceived the study. XD and CJ were in charge of overall direction and planning, and supervised the project. MV-C, SB, and XD contributed to the investigation and collection of the data. MV-C, LM-M, XD, and AD contributed to the interpretation of the results. LM-M performed the statistical analysis. MV-C took the lead in the initial manuscript, supervised by XD. SB, AD, CJ, and SR provided critical feedback and helped shape the research, analysis, and manuscript. All authors approved the final version of the manuscript.

### Conflict of Interest Statement

XD reports grants from Ministry of health, non-financial support from Masimo Inc., during the conduct of the study; personal fees, and non-financial support from Chiesi Pharmaceuticals, outside the submitted work. CJ reports grants from the Ministry of Health during the conduct of the study. The remaining authors declare that the research was conducted in the absence of any commercial or financial relationships that could be construed as a potential conflict of interest.

## References

[1] Kumar P Denson SE Mancuso TJ. Premedication for non-emergency endotracheal intubation in the neonate. Pediatrics (2010) 125:608–15. 10.1542/peds.2009-2863 20176672

[2] Friesen RH Honda AT Thieme RE. Changes in anterior fontanel pressure in preterm neonates during tracheal intubation. Anesth Analg. (1987) 66:874–8. 3619094

[3] Durrmeyer X Daoud P Decobert F Boileau P Renolleau S Zana-Taieb E. Premedication for neonatal endotracheal intubation: results from the epidemiology of procedural pain in neonates study. Pediatr Crit Care Med J Soc Crit Care Med World Fed Pediatr Intensive Crit Care Soc. (2013) 14:e169–75. 10.1097/PCC.0b013e3182720616 23439457

[4] Muniraman HK Yaari J Hand I. Premedication use before non-emergent intubation in the newborn infant. Am J Perinatol. (2015) 32:821–4. 10.1055/s-0034-1543987 25607227

[5] Barrington K. Premedication for endotracheal intubation in the newborn infant. Paediatr Child Health (2011) 16:159–71. 22379381 10.1093/pch/16.3.159 PMC3077307

[6] Durrmeyer X Dahan S Delorme P Blary S Dassieu G Caeymaex L. Assessment of atropine-sufentanil-atracurium anaesthesia for endotracheal intubation: an observational study in very premature infants. BMC Pediatr. (2014) 14:120. 10.1186/1471-2431-14-120 24886350 PMC4028002

[7] Ghanta S Abdel-Latif Me Lui K Ravindranathan H Awad J Oei J. Propofol compared with the morphine, atropine, and suxamethonium regimen as induction agents for neonatal endotracheal intubation: a randomized, controlled trial. Pediatrics (2007) 119:e1248–55. 10.1542/peds.2006-2708 17485450

[8] Welzing L Kribs A Eifinger F Huenseler C Oberthuer A Roth B. Propofol as an induction agent for endotracheal intubation can cause significant arterial hypotension in preterm neonates. Paediatr Anaesth. (2010) 20:605–11. 10.1111/j.1460-9592.2010.03330.x 20642659

[9] Simons SH Van Der Lee R Reiss IK Van Weissenbruch MM. Clinical evaluation of propofol as sedative for endotracheal intubation in neonates. Acta Paediatr Oslo Nor. (2013) 102:e487–92. 10.1111/apa.12367 23889264

[10] Hunt RW Evans N Rieger I Kluckow M. Low superior vena cava flow and neurodevelopment at 3 years in very preterm infants. J Pediatr. (2004) 145:588–92. 10.1016/j.jpeds.2004.06.056 15520755

[11] Kluckow M Evans N. Low superior vena cava flow and intraventricular haemorrhage in preterm infants. Arch Dis Child Fetal Neonatal Ed. (2000) 82:F188–94. 10.1136/fn.82.3.F188 10794784 PMC1721081

[12] Lou HC Lassen NA Friis-Hansen B. Impaired autoregulation of cerebral blood flow in the distressed newborn infant. J Pediatr. (1979) 94:118–21. 758388 10.1016/s0022-3476(79)80373-x

[13] Tsuji M Saul JP du Plessis A Eichenwald E Sobh J Crocker R. Cerebral intravascular oxygenation correlates with mean arterial pressure in critically ill premature infants. Pediatrics (2000) 106:625–32. 11015501 10.1542/peds.106.4.625

[14] Dempsey EM Barrington KJ. Diagnostic criteria and therapeutic interventions for the hypotensive very low birth weight infant. J Perinatol Off J Calif Perinat Assoc. (2006) 26:677–81. 10.1038/sj.jp.7211579 16929346

[15] Groves AM Kuschel CA Knight DB Skinner JR. Relationship between blood pressure and blood flow in newborn preterm infants. Arch Dis Child Fetal Neonatal Ed. (2008) 93:F29–32. 10.1136/adc.2006.109520 17475696

[16] Dempsey EM Hazzani FAL Barrington KJ. Permissive hypotension in the extremely low birthweight infant with signs of good perfusion. Arch Dis Child Fetal Neonatal Ed. (2009) 94:F241–4. 10.1136/adc.2007.124263 19174413

[17] Thewissen L Caicedo A Lemmers P Van Bel F Van Huffel S Naulaers G. Measuring near-infrared spectroscopy derived cerebral autoregulation in neonates: from research tool toward bedside multimodal monitoring. Front Pediatr. (2018) 6:117. 10.3389/fped.2018.00117 29868521 PMC5960703

[18] Myers DE Anderson LD Seifert RP Ortner JP Cooper CE Beilman GJ. Non-invasive method for measuring local hemoglobin oxygen saturation in tissue using wide gap second derivative near-infrared spectroscopy. J Biomed Opt. (2005) 10:034017. 10.1117/1.1925250 16229661

[19] Hyttel-Sorensen S Pellicer A Alderliesten T Austin T Van Bel F Benders M. Cerebral near infrared spectroscopy oximetry in extremely preterm infants: phase ii randomised clinical trial. BMJ (2015) 350:g7635. 10.1136/bmj.g7635 25569128 PMC4283997

[20] van den Berg E Lemmers PM Toet MC Klaessens JH van Bel F. Effect of the “InSurE” procedure on cerebral oxygenation and electrical brain activity of the preterm infant. Arch Dis Child Fetal Neonatal Ed. (2010) 95:F53–8. 10.1136/adc.2008.156414 19679893

[21] Bertini G Coviello C Gozzini E Bianconi T Bresci C Leonardi V. Change of Cerebral oxygenation during surfactant treatment in preterm infants: “LISA” versus “InSurE” procedures. Neuropediatrics (2017) 48:98–103. 10.1055/s-0037-1598647 28245505

[22] Smits A Thewissen L Caicedo A Naulaers G Allegaert K. Propofol dose-finding to reach optimal effect for (Semi-)elective intubation in neonates. J Pediatr. (2016) 179:54–60.e9. 10.1016/j.jpeds.2016.07.049 27597733

[23] Thewissen L Caicedo A Dereymaeker A Van Huffel S Naulaers G Allegaert K. Cerebral autoregulation and activity after propofol for endotracheal intubation in preterm neonates. Pediatr Res. (2018) 8:719–25. 10.1038/s41390-018-0160-3 30201953

[24] Durrmeyer X Breinig S Claris O Tourneux P Alexandre C Saliba E. Effect of atropine with propofol vs. atropine with atracurium and sufentanil on oxygen desaturation in neonates requiring non-emergency intubation: a randomized clinical trial. JAMA (2018) 319:1790–801. 10.1001/jama.2018.3708 29715354 PMC6583687

[25] Naulaers G Meyns B Miserez M Leunens V Van Huffel S Casaer P. Use of tissue oxygenation index and fractional tissue oxygen extraction as non-invasive parameters for cerebral oxygenation. A validation study in piglets. Neonatology (2007) 92:120–6. 10.1159/000101063 17377413

[26] Dix LML van Bel F Lemmers PMA. Monitoring cerebral oxygenation in neonates: an update. Front Pediatr. (2017) 5:46. 10.3389/fped.2017.00046 28352624 PMC5348638

[27] Michelet D Arslan O Hilly J Mangalsuren N Brasher C Grace R. Intraoperative changes in blood pressure associated with cerebral desaturation in infants. Paediatr Anaesth. (2015) 25:681–8. 10.1111/pan.12671 25929346

[28] Koch HW Hansen TG. Perioperative use of cerebral and renal near-infrared spectroscopy in neonates: a 24-h observational study. Paediatr Anaesth. (2016) 26:190–8. 10.1111/pan.12831 26725989

[29] Kooi EMW van der Laan ME Verhagen EA Van Braeckel KN Bos AF. Volume expansion does not alter cerebral tissue oxygen extraction in preterm infants with clinical signs of poor perfusion. Neonatology (2013) 103:308–14. 10.1159/000346383 23548640

[30] Moher D Hopewell S Schulz KF Montori V Gøtzsche PC Devereaux PJ. CONSORT 2010 explanation and elaboration: updated guidelines for reporting parallel group randomised trials. BMJ (2010) 340:c869. 10.1136/bmj.c869 20332511 PMC2844943

[31] Petrova A Mehta R. Regional tissue oxygenation in association with duration of hypoxaemia and haemodynamic variability in preterm neonates. Arch Dis Child Fetal Neonatal Ed. (2010) 95:F213–9. 10.1136/adc.2009.161604 19948524

[32] Chandrasekharan P Nrusimha A Rawat M Lakshminrusimha S. Using paralytic as part of premedication for elective intubation of premature neonates may result in transient impairment of ventilation. Am J Perinatol. (2018) 35:1127–30. 10.1055/s-0038-1635092 29510421

[33] Fleck T Schubert S Ewert P Stiller B Nagdyman N Berger F. Propofol effect on cerebral oxygenation in children with congenital heart disease. Pediatr Cardiol. (2015) 36:543–9. 10.1007/s00246-014-1047-7 25311762

[34] Vanderhaegen J Naulaers G Van Huffel S Vanhole C Allegaert K. Cerebral and systemic hemodynamic effects of intravenous bolus administration of propofol in neonates. Neonatology (2010) 98:57–63. 10.1159/000271224 20051696

[35] Alderliesten T Lemmers PM Smarius JJ Van De Vosse Re Baerts W Van Bel F. Cerebral oxygenation, extraction, and autoregulation in very preterm infants who develop peri-intraventricular hemorrhage. J Pediatr. (2013) 162:698–704.e2. 10.1016/j.jpeds.2012.09.038 23140883

[36] König K Casalaz DM Burke EJ Watkins A. Accuracy of non-invasive blood pressure monitoring in very preterm infants. Intensive Care Med. (2012) 38:670–6. 10.1007/s00134-012-2499-y 22392028

[37] Wong FY Leung TS Austin T Wilkinson M Meek JH Wyatt JS. Impaired autoregulation in preterm infants identified by using spatially resolved spectroscopy. Pediatrics (2008) 121:e604–11. 10.1542/peds.2007-1487 18250118

