# Supplementary material for: Cerebral Oxygenation During Neonatal Intubation–Ancillary Study of the Prettineo–Study
Source: Front Pediatr. 2019 Mar 1;7:40. doi: 10.3389/fped.2019.00040 (PMC6407664; doi:10.3389/fped.2019.00040)
Supplement: Supplementary file 1 [file Data_Sheet_1.PDF]

Cerebral oxygenation during neonatal intubation- ancillary study of the PRETTINEO – Study

Supplement : Reasons for prolonged interruption of the main trial and elaboration of the present ancillary study

A prolonged interruption occurred for the main trial between August 2014 and March 2016 after the first drug manufacturing company (Amatsi, Saint-Augustin, France) unilaterally decided to stop the production with short notice. Another company capable of producing appropriately masked placebo propofol kits was found only in Switzerland (Baccinex, Courroux, Switzerland) after 2 months. Because regulatory rules did not permit the export of opioids or anesthetics across borders, the sponsor asked a complementary French manufacturing company (Theradis Pharma, Cagnes-sur-Mer, France) to produce all items of the study kits, except the placebo for propofol, which was produced in Switzerland. The numerous issues in coordinating the production and obtaining adequate authorizations and guarantees from all parties explain the 19-month interruption. During this interruption, precisely in September 2015, the present ancillary study was elaborated and designed to describe cerebral NIRS profiles.
