# Supplementary figures and images for: Cerebral Oxygenation During Neonatal Intubation–Ancillary Study of the Prettineo–Study
Source: Front Pediatr. 2019 Mar 1;7:40. doi: 10.3389/fped.2019.00040 (PMC6407664; doi:10.3389/fped.2019.00040)

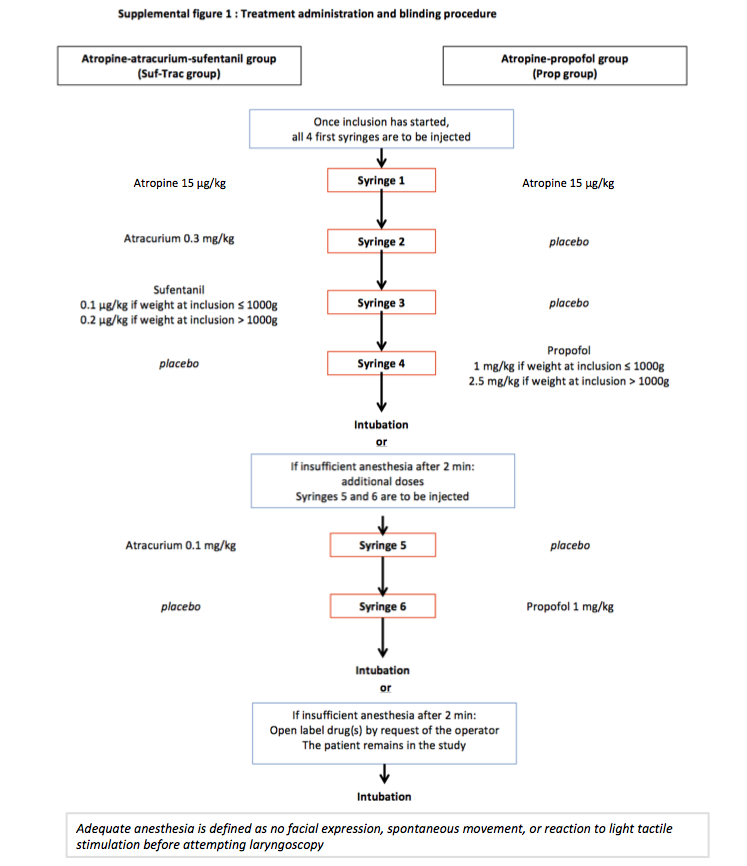

Supplement: Supplementary file 2 [file Image_1.TIFF]
